# Population pharmacokinetics model of pyrazinamide to optimize tuberculosis treatment: An interethnic cohort study of diabetes mellitus effect on drug exposure

**DOI:** 10.1371/journal.pone.0340133

**Published:** 2026-01-29

**Authors:** Rannissa Puspita Jayanti, Yong-Soon Cho, Soedarsono Soedarsono, Hyo-Jung Kim, Jiyeon Kang, Jehun Kim, Jee Youn Oh, Bo Hyoung Kang, Jick Hwan Ha, Jin-Woo Kim, Ni Made Mertaniasih, Tutik Kusmiati, Ariani Permatasari, Rika Yuliwulandari, Ryunha Kim, Hyeon-Jeong Seong, Jong-Lyul Ghim, Dong-Hyun Kim, Jae-Gook Shin, on behalf of the cPMTb

**Affiliations:** 1 Center for Personalized Precision Medicine of Tuberculosis, Inje University College of Medicine, Busan, Republic of Korea; 2 Department of Pharmacology and PharmacoGenomics Research Center, Inje University College of Medicine, Busan, Republic of Korea; 3 Department of Clinical Pharmacology, Inje University Busan Paik Hospital, Busan, Republic of Korea; 4 Sub-Pumonology Department of Internal Medicine, Faculty of Medicine, Hang Tuah University, Surabaya, Indonesia; 5 Department of Pulmonology and Respiratory Medicine, Faculty of Medicine, Universitas Airlangga, Surabaya, Indonesia; 6 Institute of Tropical Disease, Universitas Airlangga, Surabaya, Indonesia; 7 Dr. Soetomo General Hospital, Surabaya, Indonesia; 8 Division of Pulmonology, Department of Internal Medicine, Inje University Haeundae Paik Hospital, Busan, Republic of Korea; 9 Department of Internal Medicine, Inje University Ilsan Paik Hospital, Goyang, Republic of Korea; 10 Pulmonary Division, Department of Internal Medicine, Kosin University Gospel Hospital, Busan, Republic of Korea; 11 Division of Pulmonology, Department of Internal Medicine, Korea University Guro Hospital, Seoul, Republic of Korea; 12 Division of Pulmonology, Department of Internal Medicine, Dong-A University Hospital, Busan, Republic of Korea; 13 Division of Pulmonary and Critical Care Medicine, Department of Internal Medicine, Incheon St. Mary’s Hospital, College of Medicine, The Catholic University of Korea, Seoul, Republic of Korea; 14 Department of Internal Medicine, Uijeongbu St. Mary’s Hospital, College of Medicine, The Catholic University of Korea, Seoul, Republic of Korea; 15 Departement of Clinical Microbiology, Faculty of Medicine, Universitas Airlangga, Surabaya, Indonesia; 16 Faculty of Medicine, University of Pembangunan Nasional Veteran Jawa Timur, Surabaya, Indonesia; University of Madras, INDIA

## Abstract

Diabetes mellitus (DM), a common comorbidity in tuberculosis (TB) patients, can alter the pharmacokinetics (PK) of TB drugs. Additionally, clinical and demographic differences may contribute to interethnic PK variability. However, current WHO-recommended doses for pyrazinamide (PZA) do not account for those factors. We aimed to evaluate factors related to interindividual variability (IIV) and interethnic differences in the PK of PZA between Korean and Indonesian patients with TB. Demographics, clinical characteristics, and PZA concentrations obtained from hospitals in both countries were used for model establishment. Population PK models were developed using the nonlinear mixed-effect method. A Monte Carlo simulation was performed sequentially to evaluate optimal PZA dosing strategies. A one-compartment model with allometric scaling adequately described the PK of PZA. Internal validation of the model showed good performance. No significant interethnic differences in PK parameters were observed. There were 23% and 26% increases in apparent clearance (CL/F) of Indonesian patients with DM (CL/F 3.18 L/h) and Korean patients aged > 60 years with DM (CL/F 3.5). PZA doses of 1000–1250 mg for patients of bodyweight < 40 kg and 1250 mg for older patients with DM in this weight band had a 90% probability of attaining the target AUC_0–24_ ≥ 363 mg·h/L These findings indicate that DM strongly influenced IIV, particularly in older patients. We recommend higher PZA doses for patients <40 kg and older patients with DM in this weight band. Our model provides a basis for implementing model-informed precision dosing-based therapeutic drug monitoring in both ethnicities.

## Introduction

In 2022, tuberculosis (TB) caused 1.47 million deaths globally [1]. Despite major improvements in therapeutic regimens and treatment outcomes, TB has remained a global health threat [2]. Indonesia has a high TB burden, with an estimated incidence of 312 per 100,000 population, while South Korea has an incidence of 49 per 100,000 population, the highest among Organization for Economic Co-operation and Development countries [1,3]. TB eradication demands multinational collaboration and improvement in TB management [2]. One approach to improve the treatment of TB is to optimize the dosing of current drugs.

Pyrazinamide (PZA) is an important component of first-line anti-TB therapy due to its excellent sterilizing activity [4]. In the liver, PZA is metabolized primarily by amidase to produce pyrazinoic acid (PA), which may be further oxidized by xanthine oxidase (XO) to generate 5-hydroxy-pyrazinoic acid (5-OH-PA) [5]. The pharmacokinetics (PK) of PZA has wide interindividual variability (IIV), with Xu et al. reporting up to sevenfold differences in steady-state area under the curve (AUC) among patients receiving weight-banded doses [6]. This variability may arise from both clinical factors and interethnic differences [7–9]. Ethnicity can influence PK profiles through genetic polymorphisms affecting drug-metabolizing enzymes and transporters, as well as environmental factors such as diet or co-administered medicines [10,11]. For example, polymorphisms in XO may result in differences of XO activity across populations, leading to ethnic variability in PZA metabolism [5]. Understanding these effects is important, as PK differences can alter drug exposure, therapeutic response, and risk of toxicity, potentially requiring dose adjustment in specific populations.

Furthermore, comorbidities such as diabetes mellitus (DM) are clinically relevant covariates. DM has been widely reported to hamper successful TB treatment [12,13]. DM reduces PZA exposure by increasing clearance through enhanced XO activity and has been linked to poor treatment outcomes [8,14]. Therefore, response should be monitored closely during treatment in TB patients with DM.

Other factors, including bodyweight, co-infection with human immunodeficiency virus (HIV), sex, and concomitant medications, can also influence PZA PK, possibly necessitating dose adjustment [15]. Information on anti-TB drugs’ PK/pharmacodynamics (PK/PD) parameters coupled with therapeutic drug monitoring (TDM) can support improvement in treatment [16]. Although not commonly used clinically, model-informed precision dosing (MIPD)-based TDM has shown promise [17]. MIPD-based TDM applies population PK to predict the individual concentrations of patients and evaluate covariates that significantly influence IIV in drug exposure [17]. Population PK models based on a representative population are needed to provide appropriate dosing recommendations considering potential interethnic differences in PK [15].

Current anti-TB dosing regimens are primarily based on PK/PD data from studies involving Caucasian populations [18]. Several population PK models of PZA have been developed in African and Caucasian cohorts, demonstrating variability in PK parameter estimates even within the same ethnic group [8,19–21]. However, the appropriateness of applying these dosing recommendations to other ethnic groups remains uncertain. Few population PK studies of PZA have been conducted in Asian populations, and none have assessed whether variability in PK parameters differs among Asian ethnic groups [19,22]. Understanding interethnic differences in PZA PK among Asian populations is important to informing optimal dosing strategies. Therefore, we aimed to evaluate the interethnic differences in PK parameters of PZA between Indonesian and Korean TB patients, identify covariates significantly related to the IIV of PZA exposure, and provide optimal dose suggestions for both ethnicities.

## Materials and methods

### Ethical approval and patient consent statement

This study was performed in accordance with the Declaration of Helsinki and the guidelines of each participating institution with the clinical trial number NCT05280886. The institutional review board (IRB) of each clinical site involved in the study reviewed and approved the ethical clearance and study protocol. In Korea, the host institution was Inje University Busan Paik Hospital (IRB No. 2018-02-181), and in Indonesia, the study was approved by the IRB of Dr. Soetomo General Academic Hospital (IRB No. 0641/KEPK/IV/2023). Written informed consent was obtained from all patients participating in the study before any study-related procedure was conducted. Additional information related to the ethics approval and study design is provided in S1 File.

### Study data and population

The Center for Personalized Precision Medicine of Tuberculosis (cPMTb) cohort was multinational-prospective TB cohort study conducted across 22 hospitals in Korea and one hospital in Indonesia. This cohort was established to support personalized pharmacotherapy strategies for TB patients and collects clinical information and biological samples to enable the development of population pharmacokinetic (PK) models. The current study utilized data from this cohort as part of ongoing research projects. Patients included in this study were those recruited between August 20^th^, 2018, and November 3^rd^, 2021. For research purposes, data was accessed from our database on November 17^th^, 2021. All data were securely stored in a centralized database (https://smart.cpmtb.kr/#/cohort/status) that provides real-time summaries and restricted access for authorized users, ensuring confidentiality and ethical compliance. Patients aged ≥ 18 years diagnosed with drug-susceptible TB and receiving a PZA-based regimen for at least 2 weeks were enrolled in the study. The PZA dosing regimen followed the current WHO guidelines for TB treatment [18]. Patients who were nonadherent, pregnant, and diagnosed as multi-drug resistant were excluded. The demographic characteristics of the enrolled patients, comorbidities, relevant comedications, and laboratory testing results were recorded.

For this study, we used data from 703 Korean TB patients and 186 Indonesian TB patients. In total, 77 plasma concentration samples from Korean patients (11% of samples, with each patient contributing one sample) and 40 samples from Indonesian patients (also 11% of samples, as 20 patients contributed two samples each) were below the lower limit of quantification (BLLoQ) and removed from the analysis due to initial recruitment batch when sampling workflows were being standardized. Additionally, 22 Korean patients and 6 Indonesian patients with outlier concentrations identified through graphical and clinical verification were excluded. We evaluated robustness to BLLoQ handling by refitting the same base population PK model with BLLoQ observations included vs excluded, then comparing PK parameter estimates, precision, error-model parameters, and GOF diagnostics. To evaluate the interethnic effects without bias, we matched the body weight and age of the enrolled patients between ethnicities with the maximum difference of body weight and age were 5 kg and 5 years old, respectively. The Indonesian subjects were used as the reference group in subjects matching selection. The raw data and sample inclusion workflow are illustrated in S1 and S2 Figs, respectively.

### Sampling strategy of pyrazinamide

Blood samples (5 mL) were collected from participants at random times between 0–24 h after the last PZA administration and kept in heparin-containing tubes. One sample was obtained from outpatients, while at least two samples were taken from those hospitalized. A portion of 3-mL blood sample was centrifuged at 2000 × *g* at 4°C for 10 min to separate the plasma. The plasma was collected immediately after sampling and stored at a temperature below −80°C until the bioanalysis was conducted.

### Quantification of plasma pyrazinamide

A high-performance liquid chromatography–electrospray ionization–tandem mass spectrometry was used to measure the plasma concentration of PZA following our validated method [23]. Gradient elusion on a reverse-phase dC18 column separated the plasma samples. Acetonitrile was used to precipitate the protein from plasma. Detection was performed on the QTRAP 4000 mass spectrometer (Applied Biosystems, Foster City, CA, USA) equipped with a Turbolon-Spray source. The calibration range was 2.0–80.0 mg/L with a correlation coefficient of 0.9988. The LLOQ of PZA using this method was 2.0 μg/mL. The intraday and interday accuracy ranges were 93.3–109.4% and 93.3–109.4%, respectively. The analysis of PZA concentration was centralized in Korea to exclude the possibility of different methods of bioanalysis affecting the result of estimated PK parameters.

### Population pharmacokinetics modelling and dose simulation

Population PK analysis was performed using NONMEM software (version 7.4.1; ICON Development Solutions, Ellicott City, MD, USA), and PK parameters were calculated using first-order conditional estimation via ɛ-η interaction. R software (version 4.1.0; R Development Core Team, Vienna, Austria) was used to analyze the data and generate graphs. The PZA plasma concentrations below the LLOQ were excluded. All the enrolled subjects were used for model establishment. The dataset used for the PZA population PK analysis is provided as S2 File. Several structural models were evaluated: one- and two- compartment disposition kinetics with first order elimination and several approaches for examining the possibility of an absorption delay using different absorption models, i.e., lag-time model, sequential zero- and first-order absorption models, and transit compartment model. Interindividual variability (IIV) was assumed to follow a log-normal distribution. Allometric scaling of either body weight or lean body weight (LBW) was tested on apparent clearance (CL/F) and central volume of distribution (Vd/F). After comparing estimated exponents with standard allometric exponents, LBW-based scaling using fixed exponents of 0.75 for CL/F and 1 for Vd/F was incorporated due to significant improvement in model fit and stability [24]. Additive, proportional, and combined error models were tested to describe the residual error. After the establishment of the base model, a correlation matrix plot was generated to identify the potential significant covariates. A likelihood ratio test was used for inclusion of covariates. Covariates were applied to the model using a stepwise selection procedure consisting of forward inclusion and backward elimination. For forward inclusion, covariates were added sequentially to the model if their inclusion resulted in a decrease in objective function value (OFV) of ≥ 3.84 (p < 0.05) and they were physiologically plausible. The most statistically significant covariates were entered first, and additional covariates with p < 0.01 were added sequentially. Backward elimination was then conducted by removing each covariate individually to assess whether its exclusion caused a significant increase in OFV (ΔOFV > 7.88, p < 0.005).

Age, body weight, lean body weight, albumin, serum creatinine, estimated glomerular filtration rate (eGFR), blood urea nitrogen (BUN), AST, ALT, and total bilirubin were included as continuous covariates. Meanwhile, sex, ethnicity, DM, liver disease, and geriatric (≥ 60 years) with DM were investigated as categorical covariates of PK parameters. eGFR was calculated using the CKD-EPI 2009 equation [25]. For males with a serum creatinine ≤ 0.9 mg/dL, the equation is $eGFR=\text{1}\text{4}\text{1}\;x{\left(\frac{Scr}{\text{0}.\text{9}}\right)}^{-\text{0}.\text{4}\text{1}\text{1}}x\;{\text{0}.\text{9}\text{9}\text{3}}^{Age}$. Lean body weight (LBW) was calculated using the Boer equation [26]: LBW (male)=0.407 x weight (kg)+0.267 x height (cm)−19.2  and LBW (female)=0.252 x weight (kg)+0.473 x height (cm)−48.3. The effects of continuous covariates were explored using the power function with the following equation: $P={\left(\theta_{\text{TV}\;}\times \frac{{Cont_{COV}}_{i}}{{Cont_{COV}}_{median}}\right\}}^{\theta_{p}}\backslash )$, where $\theta_{\text{TV}\;}\;$ represents the typical value of the PK parameter (*P*), ${Cont_{COV}}_{i}$ is the value of the continuous variable for the *i*th patient, ${Cont_{COV}}_{median}$ is the median value for a continuous covariate, and $\theta_{p}$ is the exponent of the power function. On the other hand, the effects of categorical variables were tested using a similar function: $P=\theta_{i}\times \left(\text{1}+\;\theta_{i+\text{1}\;}\times {Cat\_Cov}_{i+\text{1}}\right)$. Following the previous equation, $\theta_{i\;}$ represents the estimated effect of the *i*th categorical covariate (when ${Cat\_Cov}_{i+\text{1}}$ = 0), and $\theta_{i+\text{1}\;}$ is the estimated effect of the *i* + 1th categorical variable relative to $\theta_{i}$ (when ${Cat\_Cov}_{i+\text{1}}$ = 1).

The base and final models were selected based on multiple criteria, including a significant decrease in Objective Function Value (OFV) determined by the likelihood ratio test, successful minimization, physiological plausibility of estimated parameters compared to literature values, acceptable goodness-of-fit plots, and passing the Wald’s test for reliable standard error estimation. The final model was internally validated through predictive-corrected visual predictive (pc-VPC) testing and robustness of the estimated PK, and parameters were evaluated by nonparametric bootstrap analysis. The final model was considered well validated when the mean values of the estimated parameters fell within the 95% confidence interval (CI).

Based on WHO-defined weight bands, the final PK model was used to simulate steady-state PZA concentrations achieved during the treatment of DS-TB after administration of feasible doses of 1000, 1250, 1500, 2000, 2500, and 3000 mg. Further comparisons were conducted with simulations based on WHO weight-band dosing of 800, 1200, 1600, and 2000 mg [18]. Using the final model, Monte Carlo simulations with 1,000 replications were performed on 1,000 virtual TB patients in a steady state. The demographic characteristics of these virtual patients were generated to match the study population, with continuous covariates simulated based on mean and standard deviation values, and categorical covariates allocated proportionally. For each dosing strategy, we determined the proportion of patients in each weight band achieving a target area under the concentration-time curve from 0 to 24 h (AUC_0–24_) of 363 mg·h/L, which has been associated with successful TB treatment outcomes [27]. Under different dosing regimens, we identified the optimal dose at which 90% of patients achieved the target AUC_0–24_ for PZA in the different weight bands for each ethnicity and covariate. The reported maximum concentration (C_max)_ threshold of > 60 mg/L, which is considered the upper limit of the effective therapeutic range for PZA in TDM practice, was used to evaluate the probability of toxicity for each simulated dosing regimen [28].

## Results

### Population characteristics

A total of 320 patients with 407 plasma PZA concentration measurements were used to establish the model. One hundred and sixty patients were selected for each ethnicity. Most Indonesian subjects had two sampling points, resulting in 240 PZA concentrations; and Korean subjects provided 167 concentrations. Samples below the LLoQ were excluded, as including them led to underprediction in GOF diagnostics and reduced parameter precision. The study population had a median age of 46 years (interquartile range [IQR] 31–57 years), bodyweight of 55 kg (IQR 50–59.8 kg), and lean body weight (LBW) of 45.5 kg [IQR 40.8–49.3 kg]). The proportion of male patients was 61.6%. Seventy-seven patients had DM; among them, 25 were ≥ 60 years old. The distribution of key covariates in the two ethnicities was statistically similar and considered well-matched (p > 0.05). Baseline patient characteristics are listed in Table 1.

**Table 1:** Patient demographic characteristics.

Characteristics	Total (n = 320) ^a^	Korean (n = 160)	Indonesian (n = 160)
Sex, n (%)			
Male	197 (61.6)	90 (56.2)	107 (66.9)
Female	123 (38.4)	70 (43.8)	53 (33.1)
DM, n (%)^b^			
Yes	77 (24.1)	22 (13.7)	55 (34.3)
No	243 (75.9)	138 (86.3)	105 (65.6)
Age > 60 years old, n (%) ^b^			
With DM	25 (7.8)	13 (8.1)	12 (7.5)
Without DM	295 (92.2)	147 (91.9)	148 (92.5)
Liver Impairment, n (%) ^b^			
Yes	7 (2.2)	3 (1.8)	4 (2.5)
No	313 (97.8)	157 (98.2)	156 (97.5)
DM medication, n (%) ^b^			
Insulin	32 (41.5)	0 (0.0)	32 (41.5)
Biguanide	15 (19.5)	11 (14.3)	4 (5.2)
Sulfonylurea	5 (6.4)	5 (6.4)	0 (0.0)
Dipeptidyl peptidase-IV inhibitor	7 (9.1)	7 (9.1)	0 (0.0)
Sodium-glucose co-transporter 2 inhibitor	1 (1.3)	1 (1.3)	0 (0.0)
Unknown	30 (38.9)	11 (14.2)	19 (24.7)
Dose of Z, n (%) ^c^			
500 mg	2 (0.6)	2 (1.3)	0 (0.0)
750 mg	7 (2.2)	0 (0.0)	7 (4.4)
800 mg	1 (0.3)	0 (0.0)	1 (0.6)
1000 mg	83 (25.9)	23 (14.4)	60 (37.5)
1200 mg	22 (6.9)	5 (3.1)	17 (10.6)
1250 mg	39 (12.2)	9 (5.6)	30 (18.8)
1275 mg	2 (0.6)	0 (0.0)	2 (1.3)
1500 mg	137 (42.8)	105 (65.6)	32 (20)
1600 mg	24 (7.5)	15 (9.4)	9 (5.6)
2000 mg	3 (0.9)	1 (0.6)	2 (1.3)
Regimen, n (%) ^c^			
RHZE	282 (88.1)	142 (88.8)	140 (87.5)
RHZES	18 (5.6)	0 (0.0)	18 (11.3)
HZEL	2 (0.6)	1 (0.6)	1 (0.6)
RHZ	3 (0.9)	3 (1.9)	0 (0.0)
RZEM	3 (0.9)	3 (1.9)	0 (0.0)
Others	12 (3.8)	11 (6.8)	1 (0.6)
eGFR (mL/min/1.73m^2^), median (IQR) ^d^	105.5 (97.7–121.8)	104.9 (100.4–118.9)	106.7 (91.9–123.9)
Age (y), median (IQR)	46 (31–57)	47 (31–59)	45 (30–54)
Body weight (kg), median (IQR)	55 (50–59.8)	56 (51–63)	50 (45–56.75)
Height (cm), median (IQR)	165 (160–170)	165 (160–171)	165 (160–168)
Lean Body Weight (kg), median (IQR)	45.5 (40.8–49.3)	46.2 (41.6–50.75)	44.9 (40.7–48.0)
Albumin (g/dL), – 3.8–5.3 g/dL	3.6 (3.0–4.1)	4.1 (4.1–4.4)	3.0 (2.7–3.4)
AST (U/L), – 13–33 U/L ^e^	27 (23–36.75)	24 (21.7–30)	29.0 (24.0–44.0)
ALT (U/L), – 6–27 U/L ^f^	24 (18–33)	19 (15–28)	26.0 (21.0–38.25)
Total Bilirubin (mg/dL), – 0.2–1.2 mg/dL	0.52 (0.4–0.69)	0.5 (0.48–0.73)	0.4 (0.39–0.63)
Blood Urea Nitrogen (mg/dL), – 8–22 mg/dL	10.4 (8.4–13.3)	10.4 (9.3–12.8)	10 (8–15)
Serum Creatinine (mg/dL), – 0.8–1.2 mg/dL	0.7 (0.6–0.82)	0.67 (0.61–0.79)	0.7 (0.6–0.9)

^a^ Continuous data are given as median (range) and categorical data are given as a number (%), IQR: interquartile range.

^b^ DM: Diabetes Mellitus.

^c^ R: Rifampicin, H: Isoniazid, Z: Pyrazinamide, E: Ethambutol, S: Streptomycin, L: Levofloxacin, M: Moxifloxacin, Others: other regimens that were different from the mentioned regimens.

^d^ eGFR: Estimated Glomerular Filtration Rate.

^e^ AST: Aspartate transaminase.

^f^ ALT: Alanine transaminase.

### Population pharmacokinetics model of PZA

A one-compartment model with first-order absorption–elimination with additive residual error adequately described the PK of PZA. The model estimated the IIV in both apparent clearance (CL/F) and apparent volume of distribution (Vd/F). Allometric scaling using LBW described the size effect sufficiently and significantly reduced the OFV. None of the absorption models evaluated had improved performance.

Aspartate aminotransferase (AST), albumin, and DM were significant covariates in univariate analyses (p < 0.05). AST and albumin were excluded due to large standard errors. Therefore, only DM was incorporated in the final model. The inclusion of LBW and DM in the model reduced the OFV by 25.292 (*p* < 0.001). Ethnicity did not significantly affect the CL/F and Vd/F of PZA. The age distribution of DM, as a significant covariate, differed between the two ethnicities. The Indonesian TB patients with DM tended to be < 60 years old. Most Korean TB-DM patients were older adults. Therefore, we estimated the population parameters of CL/F for each ethnicity separately ($\theta_{\text{1}}$ for Indonesians and $\theta_{\text{2}}$ for Koreans) and tested the following covariate combinations—effect of DM in both ethnicities (DM [in Indonesians] − DM [in Koreans]), older patients (≥ 60 years old) with DM in both ethnicities (OldDM [in Indonesians] − OldDM [in Koreans]), and the combination of all and old DM patients for each ethnicity independently (DM [in Indonesians] − OldDM [in Koreans] and OldDM [in Indonesians]−DM [in Koreans]). Model performance was significantly improved by estimating the population parameters of CL/F for each ethnicity separately and using the combination of DM-DM and DM-OldDM in the CL/F of Indonesian and Korean individuals, as detailed in S1 Table. Based on our model of PZA in the Korean population [29], as well as the clinical characteristics of DM in each population, we included DM (in Indonesians) − OldDM (in Koreans) as covariates of CL/F in the final model.

The effect of DM on the CL/F of Indonesian and Korean patients was 0.226 and 0.252, respectively. The typical population parameters of CL/F were estimated as 3.2 L/h for Indonesian patients without DM and 3.5 L/h for younger Korean patients with DM. CL/F estimates for Indonesian and older Korean patients with DM were 3.88 and 4.38 L/h, respectively; the estimated Vd/F and Ka values were 53.4 L and 2.1 h^−1^, respectively. The estimated PK parameters of PZA are shown in Table 2. Despite the different sampling frequencies between two ethnicities, %RSEs were similar across both ethnicities (4.7% vs 4.5%), and bootstrap 95% CIs were narrow and overlapping, indicating stable estimation for both subgroups.

**Table 2:** Population PK parameter estimates of PZA for the final model and bootstrap results.

Parameters ^a^	Typical Value	Bootstrap results	
	(%RSE) (% η-shrinkage) ^b^	Median	95%CI ^c^
**Fixed-effect parameters** ^**d**^			
CL/F_Indonesian_; = $\theta_{\text{1}},\;\theta_{\text{1}}$	3.18 (4.7)	3.18	2.9–3.5
CL/F_Korean_; = $\theta_{\text{2}},\;\theta_{\text{2}}$	3.5 (4.5)	3.5	3.2–3.8
CL/F; DM = $\theta_{\text{1}}\;\times \left(\text{1}+\;\theta_{\text{3}}\right),\;\theta_{\text{3}}\;$	0.23 (41.6)	0.23	0.07–0.48
CL/F; Old DM = $\theta_{\text{2}}\times \left(\text{1}+\;\theta_{\text{4}}\right),\;\theta_{\text{4}}$	0.26 (42.3)	0.26	0.06–0.5
Vd/F (L) = $\theta_{\text{5}},\;\theta_{\text{5}}$	52.8 (6.6)	52.7	46.2–60.6
Ka (h^-1^) = $\theta_{\text{6}},\;\theta_{\text{6}}$	2.0 (15)	2.05	1.3–2.6
**Interindividual variability**			
$\omega^{\text{2}}$; CL/F_Indonesian_ (%)	20.5 (9.7) (35.9)	20.3	15.4–26.4
$\omega^{\text{2}}$; CL/F_Korean_ (%)	5 (19.6) (56.9)	5	2.3–8.4
$\omega^{\text{2}}$; Vd/F (%)	23.6 (12.7) (24.6)	23.4	16.8–32.0
$\omega^{\text{2}}$; Ka (%)	0 (FIX)	–	–
**Residual variability**			
Additive	1.29 (14.6)	1.28	0.92–1.7

^a^ CL/F, apparent clearance; Vd/F, apparent volume of distribution; Ka, absorption rate constant; $\omega^{\text{2}}$, variance of interindividual variability.

^b^ % RSE, relative standard error [(SE/mean) x 100%]; % η-shrinkage, η-shrinkage = (1-SD(η)/ω) x 100%, where η are the between individual variation terms and ω is the population model estimate of the standard deviation in η.

^c^ CI, confidence interval.

^d^ Allometric scaling was applied to the CL/F and Vd/F data, and typical values reported here refer to the typical patient, with lean body weight of 45 kg.

**Fig 1:**
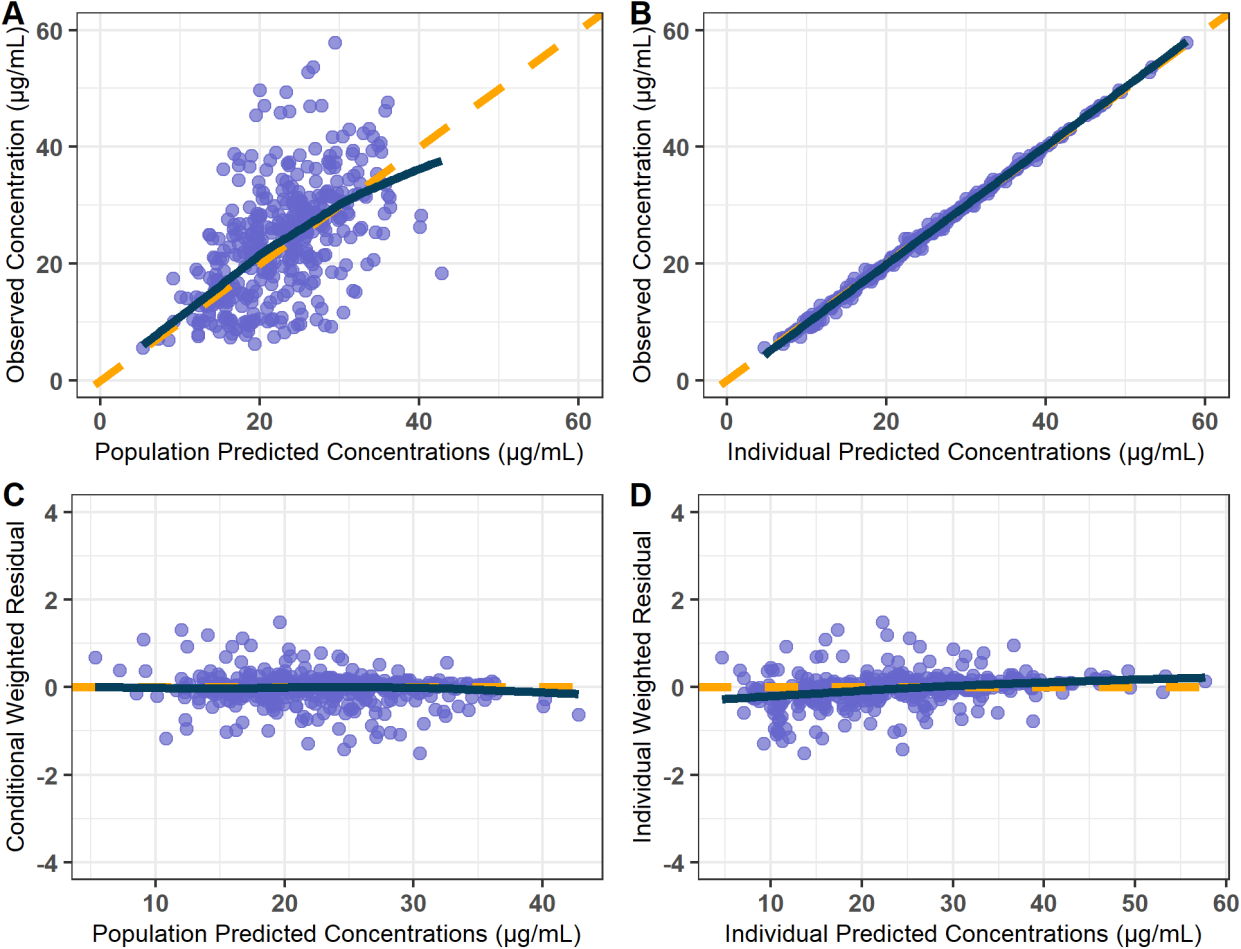
Goodness-of-fit plots of the final model. **(A)** Observed versus population predicted concentrations. **(B)** Observed versus individual predicted concentrations. **(C)** Concentration weighted residuals versus population predicted population. **(D)** Individual weighted residuals versus individual predicted concentrations. Open purple circles represent the plasma concentrations of PZA, and solid dark blue lines represent locally weighted least-squares regression according to plasma concentration.

**Fig 2:**
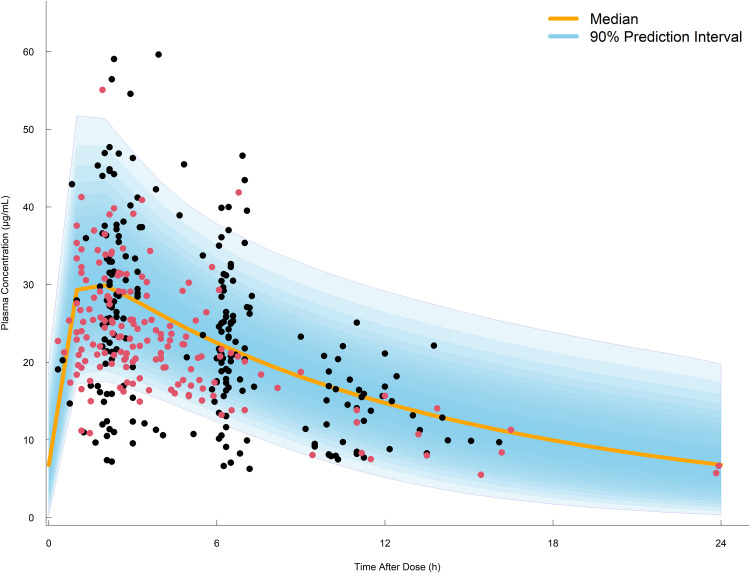
Prediction-corrected visual predictive checks. The black and red dots represent the observed PZA concentrations in Indonesian and Korean, respectively. The solid orange line represents the median predicted concentration. The shaded area represents the 90% prediction interval of the simulated PZA concentrations based on the final model.

The GOF plot of the final model is shown in Fig 1. The GOF plots showed that the observed and predicted concentrations were evenly distributed around the line of identity without trends, and most of the predicted concentrations were distributed within two standard deviations. Therefore, the structure and residual error of the final model for population and individual predictions were considered appropriate and without significant bias. Predictive-corrected visual predictive check (pc-VPC) testing also indicated the model to have good predictive capability (Fig 2). Simulated 90% prediction interval adequately captured the observed median and majority of observed data, indicating the good fit of the final model. Additionally, stratified VPCs based on key covariates can be seen in S3 and S4 Figs, further supporting the model’s good predictive performance across relevant subgroups. Parameter estimates obtained by bootstrapping analysis fell within the 95% CI and showed concordance with the results of the final model, reflecting its stability and reproducibility.

### Bayesian estimation of pyrazinamide pharmacokinetics parameters

**Fig 3:**
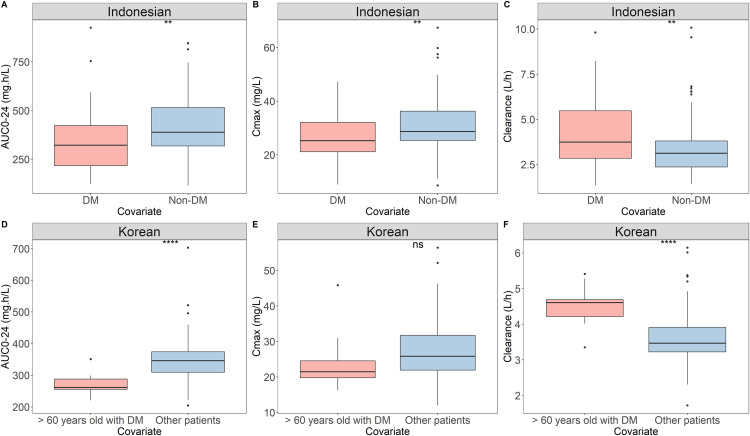
Relationships of DM and old DM with PK parameters of each ethnicity. **(A)** Area under concentration of 0 to 24 hours between DM and non-DM patients in Indonesian ethnic. **(B)** Maximum concentration between DM and non-DM patients in Indonesian ethnic. **(C)** Apparent clearance between DM and non-DM patients in Indonesian ethnic. **(D)** Area under concentration of 0 to 24 hours between old DM and other patients in Korean ethnic. **(E)** Maximum concentration between old DM and other patients in Korean ethnic. **(F)** Apparent clearance between old DM and other patients in Korean ethnic. Box plot showing the interquartile range of each PK parameter. The groups are represented as follows: pink, DM in Indonesian, and old DM in Korean; blue, non-DM in Indonesian, and other patients in Korean. The straight line in the upper part of the box plot represents the t-test results. **** P < 0.001, ns: non-significant). DM: diabetes mellitus, old DM: ≥ 60 years old patients with DM, Other patients: patients who aged < 60 years old with or without DM, PZA: Pyrazinamide, PK: pharmacokinetics.

The CL/F and Vd/F values of the ethnicities are shown in S5 Fig—there was no significant inter-ethnicity difference identified. Estimated AUC_0–24_ and C_max_ values, normalized to a dose of 1200 mg, according to ethnicity and covariate, are shown in Fig 3. DM status significantly affected the PK parameters of PZA in both ethnicities (p < 0.01). The estimated values of PK parameters of PZA between ethnicities and covariates are presented in S2 Table. Using Bayesian forecasting, the median estimated AUC_0–24_ values for Indonesian TB patients with and without DM were 322.2 mg·h/L (interquartile range [IQR] 216.8–423.4 mg·h/L) and 388.6 mg·h/L (IQR 318.1–515.4 mg·h/L), respectively. The median C_max_ values were 25.27 mg/L (IQR 21.13–32.09 mg/L) and 28.70 mg/L (IQR 25.34–36.28 mg/L) for Indonesian patients with and without DM, respectively. The median AUC_0–24_ values for older Korean TB patients with DM and other patients who did not belong to this group were 261.4 mg·h/L (IQR 255.2–287.8 mg·h/L) and 345.7 mg·h/L (IQR 309.2–374.0 mg·h/L), respectively. Among Koreans, older patients with DM and others had median C_max_ values of 21.46 mg/L (IQR 19.76–24.55 mg/L) and 25.83 mg/L (IQR: 21.90–31.72 mg/L), respectively.

For PZA, the median AUC_0–24_ values were 371.1 mg·h/L (IQR 270.2–466.9 mg·h/L) and 343.2 mg·h/L (IQR 299.6–372.5 mg·h/L) for Indonesians and Koreans, respectively. The C_max_ value of Indonesians was 27.60 mg/L (23.47–33.12 mg/L) and that of Koreans was 25.62 mg/L (21.66–31.22 mg/L); the difference was not significant. Compared to Indonesian TB-DM patients, older DM patients among Korean subjects had the lowest exposure of PZA. As expected, non-DM patients were able to achieve the target therapeutic exposure of PZA (AUC_0–24_ ≥ 363) among Indonesian subjects. Thus, the presence of DM remarkably increased the CL/F of PZA, and the effect of DM was exacerbated with advanced age in both ethnicities.

### Dose exploration

The optimal doses of PZA were predicted and compared with the WHO-recommended doses by Monte Carlo simulation. Following the AUC_0–24_ target of 363 mg·h/L, the 90% probability target attainment (PTA) of the WHO-recommended dose and the suggested dose in each weight band for each ethnicity and covariate are presented in S3 and S4 Tables, respectively. For the currently recommended dose, only 70% of patients of bodyweight < 40 kg achieved the target AUC_0–24_. Simulations showed that, overall, at least 90% of patients of bodyweight < 40 kg in both ethnicities achieved the target exposure with a dose of 1000–1250 mg. Therefore, Indonesian patients with and without DM can receive the same dose. However, the proportion of non-DM patients achieving the target exposure of PZA was higher. Interestingly, older Korean DM patients of bodyweight < 40 kg needed a dose of 1250 mg to achieve a 90% PTA. Other Korean patients of bodyweight < 40 kg required a dose of ≥ 1000 mg to attain the therapeutic target.

**Fig 4:**
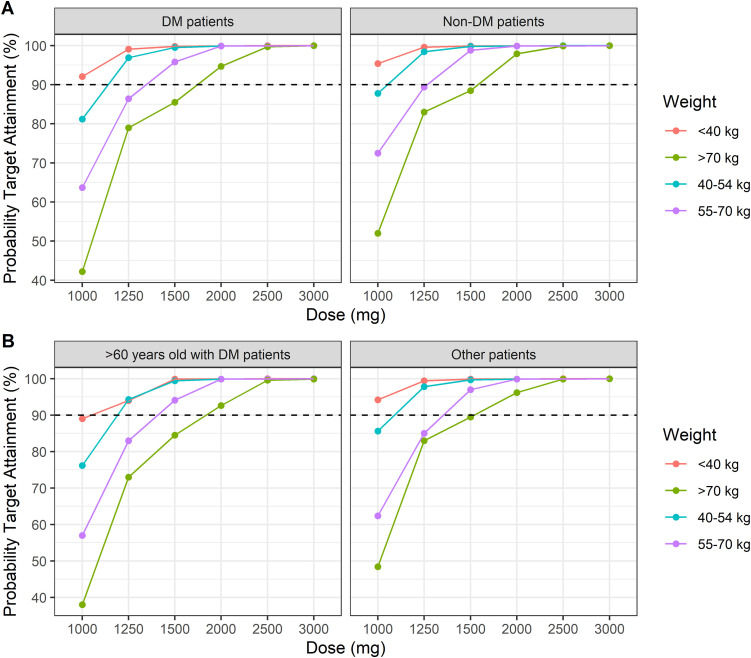
Probability of achieving a target area under the concentration-time curve from 0 to 24 h (AUC0-24) of ≥ 363 mg.h/L for the simulated PZA dosing regimens according to the different ethnicities and DM status. The WHO weight bands were represented by different colors line. Red line: < 40 kg weight band, blue line: 40-54 kg weight band, purple line: 55-70 kg weight band, and green line: > 70 kg weight band. The probability of target attainment (PTA) according to ethnicities is shown for **(A)** Indonesian; **(B)** Korean with each ethnic covariate of DM characteristics. DM: diabetes melltus, PZA: Pyrazinamide, Other patients: patients who aged < 60 years old with or without DM.

Among Indonesian patients with TB and DM, administration of 1250, 1500, and 2000 mg to patients in the 40–54, 55–70, and > 70 kg weight bands yielded 96.9%, 95.8%, and 94.7%, respectively, PTA rates. Among older Korean patients with DM, administration of 1250, 1500, and 2000 mg to those in the 40–54, 55–70, and > 70 kg weight band resulted in 94.3%, 94.1%, and 92.6% PTA rates, respectively. Therefore, the current dosing for these groups is suitable. These doses were the lowest that met the 90% PTA criteria (Fig 4).

**Fig 5:**
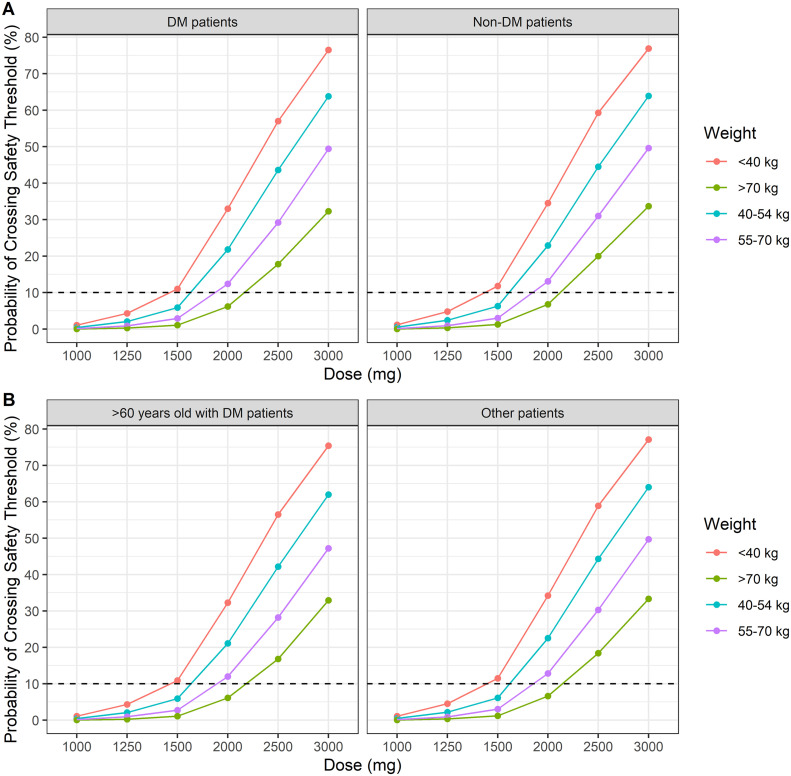
Probability of achieving maximum concentration (C_max_) of >60 mg/L for the simulated PZA dosing regimens according to the ethnicities and DM characteristics. The WHO weight bands were represented by different colors line. Red line: < 40 kg weight band, blue line: 40-54 kg weight band, purple line: 55-70 kg weight band, and green line: > 70 kg weight band. The probability of target attainment (PTA) according to ethnicities is shown for **(A)** Indonesian; **(B)** Korean with each ethnic covariate of DM characteristics. DM: diabetes melltus, PZA: Pyrazinamide, Other patients: patients who aged < 60 years old with or without DM.

To avoid drug-associated toxicity, the C_max_ should not exceed 60 mg/L. According to a dose simulation, approximately 90% of Indonesian patients of < 40, 40–54, 55–70, and > 70 kg bodyweight, irrespective of DM status, achieved a C_max_ of < 60 mg/L after once-daily doses of 1000–1250, 1250, 1500, and 2000 mg, respectively. Older and younger Korean patients with DM showed < 10% of the toxicity target with the same doses, except for older patients with DM of < 40 kg bodyweight (1250 mg). The 90% PTA values of toxicity are shown in Fig 5.

## Discussion

To the best of authors knowledge, this is the first investigation of interethnic differences in PK parameters of PZA in Asian populations by population PK analysis. A one-compartment model with first-order absorption and elimination described the PK of PZA sufficiently. Our structural model was consistent with that in previous studies [15,29]. Allometric scaling using LBW to explain the body size effect was incorporated into the CL/F and Vd/F calculation and improved the GOF of the model.

As previously reported, the CL/F of PZA increases over time [9,30]. Our estimated CL/F value of Indonesians was higher than in a prior report [19]. This could be in part because we enrolled steady-state patients whereas the prior study involved patients in early treatment. The Vd/F (53.4 L) and Ka (2.1 h^-1^) values of our model were within the reported ranges (28–62 L and 1.3–3.5 h^-1^, respectively [30].

In our study, we matched the subjects by age and body weight. Nonetheless, we did not match biological sex because most of the effect of sex on PK is a result of the different body fat compositions of males and females [31]. Since several studies have reported lower AUC_0–24_ and C_max_ values in males, primarily attributed to a higher lean-body-to-total-weight ratio [8,32,33], we accounted for this by incorporating LBW into the model, thereby addressing sex-based variability in PZA PK. We used the Indonesian data for matching samples because of the smaller sample size. We matched the sample size between ethnic groups (160 patients each) to ensure balanced comparison. While the number of concentration samples per patient differed slightly between groups, this affects only the precision of individual concentration estimates and not the population-level comparison [34,35]. Furthermore, we did not find inter-ethnicity differences in the PK of PZA. However, the CL/F and AUC_0–24_ ranges were wider in Indonesian than in Korean patients. In addition to employing a demographic matching strategy for model building, we also attempted to develop a model using the entire available data from our cohort (data not shown). This resulted in an unbalanced sample size, with Korean subjects accounting for two-thirds of the total. Our analysis revealed ethnicity to be one of significant covariates during univariate analysis but failed to be retained in the forward selection process due to high standard error. We believed our findings in the univariate analysis might be confounded by the different body weight between two ethnicities.

A recent population PK study implicated that PZA may not be influenced by ethnicity [19]. Among anti-TB drugs, INH is most likely to have inter-ethnicity differences in PK because of *N-acetyl transferase 2* (NAT2) polymorphisms [36]. Our recent studies showed twofold higher CL/F of INH in Indonesian compared to Korean within the NAT2 rapid acetylator subjects [37,38]. Although there was no difference in the PK of PZA in current studied Asian populations, the existence of differences among distinct ethnicities remains as an open field for research. Comparisons with African cohorts reported by Rockwood et al. (CL/F: 4.17 L/h), Mugabo et al. (CL/F: 4.28 L/h), Naidoo et al. (CL/F: 5.41 L/h), and Alsultan et al. (CL/F: 5.06 L/h) indicate that PZA clearance is slightly higher in African populations compared to our cohort [8,20,39,40]. Differences in PK parameters tend to increase with increasing geographic separation, due to variability in metabolic-pathway polymorphisms, diet, and environmental factors [7,41].

DM significantly affected PZA exposure in both populations. DM reportedly does not affect the CL/F of PZA in previous populations PK studies [15]. This can be explained in part by the large number of DM patients in this study. However, TB patients with DM of various ethnicities have been presented with low PZA exposure [14,42]. In this study, 55% of Korean DM patients were ≥ 60 years old, whereas 78% of the Indonesians were young adults. PZA exposure was 25.2% lower in older compared to younger patients with DM. Among the Indonesians, patients with DM had 22.6% higher exposure than those without DM.

The crisis of DM is evolving in the world, and DM increases susceptibility to TB [1]. The presence of DM has been linked to poor TB outcomes and drug resistance [14]. Indonesia has a high burden of TB in individuals 15–45 years old, and the prevalence of TB-DM increases with age [43]. By contrast, 51.6% of TB patients in Korea are ≥ 65 years old and have a high risk of DM [3,44]. Among the Indonesians, older patients with DM appeared with 40% higher CL/F than those without DM. However, the sample size was small and the inclusion of this covariate in the model increased the standard error.

DM may be associated with a reduction in the exposure to PZA among TB patients via enhanced clearance and malabsorption [14]. PZA is metabolized to 5-hydroxypyrazinoic acid in the liver by xanthine oxidase (XO) [45]. In animal and clinical studies, XO levels were markedly elevated by hyperglycemia, resulting from poorly controlled DM, and reflected by high hemoglobin A1c (HbA1c) level [11,46,47]. Elevation of XO induced by DM may have contributed to a decrease in the PZA concentration. Nevertheless, the decrease of PZA concentration potentially indicated the accumulation of PZA toxic metabolites, thereby increasing the risk of adverse drug reactions (ADRs) [48]. Additionally, an HbA1c level of > 7% is an independent risk factor for drug-resistant TB in DM patients [49]. Unfortunately, HbA1c data were not available for all the patients, particularly the Indonesians, precluding analysis of the association of HbA1c level and PZA concentration. Moreover, DM has a severe effect in older adults, who have inadequate insulin secretion and sensitivity, due to high circulating arginine vasopressin (AVP) and/or copeptin levels [50].

PZA exposure targets of ≥ 363 mg·h/L and/or C_max_ > 35 mg/L are associated with good treatment outcomes [15]. Nonetheless, our dose simulation indicated that the WHO-recommended dose achieves the target AUC_0–24_ values merely for patients of bodyweight > 40 kg. The current recommended dose of 800 mg for < 40 kg patients is likely to result in underdosing. We observed that a dose of 1000–1250 mg would be needed for such patients. Furthermore, older patients with DM of bodyweight < 40 kg required 1250 mg of PZA to achieve the target AUC_0–24_. Our result is echoed by prior reports that increased doses of PZA in low body-weight bands were required for a good therapeutic outcome [9,22]. Furthermore, use of high doses of PZA requires caution, because ADRs induced by PZA are likely to be more severe in low-bodyweight or older patients [51].

However, several critical factors must be considered when recommending higher doses. These include patient clinical conditions such as liver and renal function, comorbidities like HIV that may alter drug clearance or absorption, and concomitant medications with potential interactions [45]. Importantly, as PZA is a prodrug metabolized to pyrazinoic acid, which confers its antimycobacterial activity, increasing the dose may elevate metabolite concentrations and consequently increase the risk of hepatotoxicity or hyperuricemia [48]. Therefore, it is foremost to balance achieving adequate exposure for efficacy against the potential for dose-related adverse effects, particularly in vulnerable patients such as the elderly or those with underlying liver dysfunction [29,52]. Adherence considerations, including pill burden and formulation availability for practical dosing, and ethnic or genetic variability affecting PK, should also guide dosing decisions [53,54].

Our findings suggest the need for TDM in DM patients due to potential low exposure and the need for dose adjustment. Particularly to PZA, TDM is encouraged in cases of suspected gastrointestinal abnormalities (*e*.*g*., malabsorption, poor response) and comorbidities (*e*.*g*., HIV and DM) [9]. We also reported that PK of PZA most likely was not influenced by ethnicity. To confirm this finding, subsequent study with various ethnicities and similar study design should be performed. Additionally, those who belong to the age ≥ 60 years old with DM and < 40 kg body weight-band should be given higher dose of PZA. Otherwise, the current recommended doses are fine. A real clinical trial is needed to ensure the safety concern of high dose PZA.

The cPMTb cohort is a multinational, multicenter prospective study conducted in Korea and Indonesia, established to support personalized TB pharmacotherapy. It collects clinical data and biological samples to support the development of population PK models. Leveraging this structured real-world dataset enabled the evaluation of PK variability and covariate effects across diverse populations, enhancing the relevance of our findings to broader TB treatment settings. Despite this strength, the study had several limitations. First, our sampling strategy could reduce the precision of individual PK predictions, as previously mentioned in our recent works [55,56]. Even so, this was a compromise to reduce the hospitalization duration. Furthermore, the non-linear mixed-effects modeling applied in the analysis could effectively handle sparse data, yielding reasonable estimations for PK parameters. Second, the optimal dose exploration results were concluded based on the PK target of PZA. Optimal dose exploration based on a PK/PD target might achieve greater accuracy by accounting for individual minimum inhibitory concentration values. Finally, our current result was drawn from a small sample size from both ethnicities, follow up confirmation study with larger sample size is encouraged.

## Conclusion

We developed a population PK model of PZA using data from Korean and Indonesian TB patients to evaluate interethnic differences and the influence of clinical factors in PZA exposure. We found no significant interethnic differences in PZA PK, but DM was associated with increased CL/F in both ethnicities. Dose simulations suggested that PZA doses of 1000–1250 mg for patients with bodyweight < 40 kg, and 1250 mg for older patients with DM in this weight band, would achieve a 90% probability of attaining the target AUC_0–24_ ≥ 363 mg·h/L. Our model may facilitate MIPD-based TDM to individualize dose adjustment for Korean and Indonesian TB patients with/without DM.

## Supporting information

S1 Fig Distribution of pyrazinamide raw data concentration–time profile by ethnicity. Points are individual samples; open circles denote below lower limit of quantification observations (BLLoQ) below the dashed LLoQ line, and filled circles are quantifiable samples. Points from the same subject are connected to illustrating within-subject sampling.
(TIFF)

S2 Fig Workflow for study cohort selection and matching. This diagram illustrates the selection process from the Center for Personalized Precision Medicine of Tuberculosis (cPMTb) cohort.
(TIF)

S3 Fig Visual predictive check stratified by diabetes mellitus (DM) status. The left panel shows individuals without DM (DM == 0), and the right panel shows individuals with DM (DM == 1). Open blue circles represent observed plasma concentrations. The solid red line indicates the observed median, and the dashed red lines represent the 5th and 95th percentiles of the observed data. Shaded areas represent the 90% prediction intervals (red for median, blue for outer percentiles) from 1,000 simulations using the final model.
(TIFF)

S4 Fig Visual predictive check stratified by elderly patients with diabetes mellitus (DM) status. The left panel (DM == 0) includes all patients who are not both aged > 60 years and diagnosed with DM, while the right panel (DM == 1) includes elderly patients with DM (defined as age > 60 years with a DM diagnosis). Open blue circles represent observed plasma concentrations. The solid red line indicates the observed median, and the dashed red lines represent the 5th and 95th percentiles of the observed data. Shaded areas represent the 90% prediction intervals (red for median, blue for outer percentiles) from 1,000 simulations using the final model.
(TIFF)

S5 Fig Estimated pyrazinamide (PZA) PK parameters in comparison between enrolled ethnicities. (A) Apparent clearance among Korean-Indonesian ethnicities. (B) Apparent volume of distribution among Korean-Indonesian ethnicities Box plot showing the interquartile range of each PK parameter. The groups are represented as follows: orange, Korean; green, Indonesian. The straight line in the upper part of the box plot represents the t-test results. **** P < 0.001, ns: non-significant).
(TIFF)

S1 Table Covariate selection process of other potential models. (DOCX)

S2 Table Pharmacokinetics parameters of pyrazinamide in both ethnicities. (DOCX)

S3 Table Probability target attainment of simulated TB patients using WHO dose recommendation achieving a target AUC_0–24_ of 363 mg·h/L for both ethnicities. (DOCX)

S4 Table Probability target attainment of TB patients using simulated optimal dose achieving a target AUC_0–24_ of 363 mg·h/L for both ethnicities. (DOCX)

S1 File Human participants research checklist. (PDF)

S2 File Pyrazinamide dataset for development of the population pharmacokinetics model. (CSV)
