# Correction: Population pharmacokinetics model of pyrazinamide to optimize tuberculosis treatment: An interethnic cohort study of diabetes mellitus effect on drug exposure

**DOI:** 10.1371/journal.pone.0347490

**Published:** 2026-04-15

**Authors:** Rannissa Puspita Jayanti, Yong-Soon Cho, Soedarsono Soedarsono, Hyo-Jung Kim, Jiyeon Kang, Jehun Kim, Jee Youn Oh, Bo Hyoung Kang, Jick Hwan Ha, Jin-Woo Kim, Ni Made Mertaniasih, Tutik Kusmiati, Ariani Permatasari, Rika Yuliwulandari, Ryunha Kim, Hyeon-Jeong Seong, Jong-Lyul Ghim, Dong-Hyun Kim, Jae-Gook Shin, on behalf of the cPMTb

The Funding statement for this article is incorrect. The correct Funding statement is as follows: This work was supported by a National Research Foundation of Korea grant funded by the Ministry of Science and ICT of Republic of Korea [grant numbers No.2018R1A5A2021242, No. 2021R1F1A1056393] to J.G.S and Y.S.C, respectively; a grant from the Ministry of Research and Technology/National Agency for Research and Innovation of Indonesia [grant numbers 8478/UN3.1.1/LT/2019] to S.S; and a grant from the National Institute of Health (NIH) of Republic of Korea [grant numbers No. 2024ER200201] to Y.S.C. The funders had no role in study design, data collection and analysis, decision to publish, or preparation of the manuscript. There was no additional external funding received for this study.

## References

[1] Jayanti RP, Cho Y-S, Soedarsono S, Kim H-J, Kang J, Kim J, et al. Population pharmacokinetics model of pyrazinamide to optimize tuberculosis treatment: An interethnic cohort study of diabetes mellitus effect on drug exposure. PLoS One. 2026;21(1):e0340133. doi: 10.1371/journal.pone.0340133 41610100 PMC12854426

