# Supplementary figures and images for: Population pharmacokinetics model of pyrazinamide to optimize tuberculosis treatment: An interethnic cohort study of diabetes mellitus effect on drug exposure
Source: PLoS One. 2026 Jan 29;21(1):e0340133. doi: 10.1371/journal.pone.0340133 (PMC12854426; doi:10.1371/journal.pone.0340133)

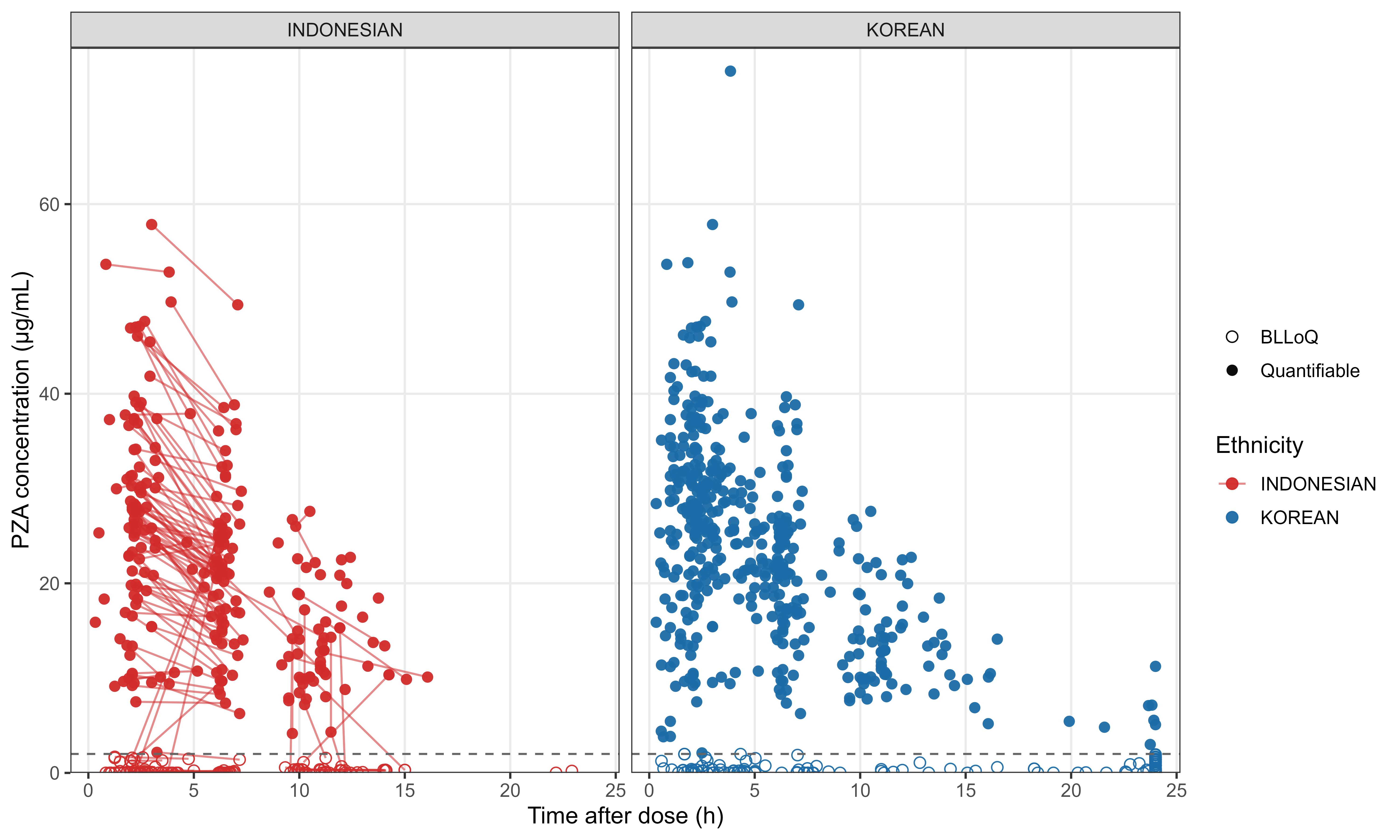

Supplement: S1 Fig — Points are individual samples; open circles denote below lower limit of quantification observations (BLLoQ) below the dashed LLoQ line, and filled circles are quantifiable samples. Points from the same subject are connected to illustrating within-subject sampling. (TIFF) [file pone.0340133.s001.tiff]

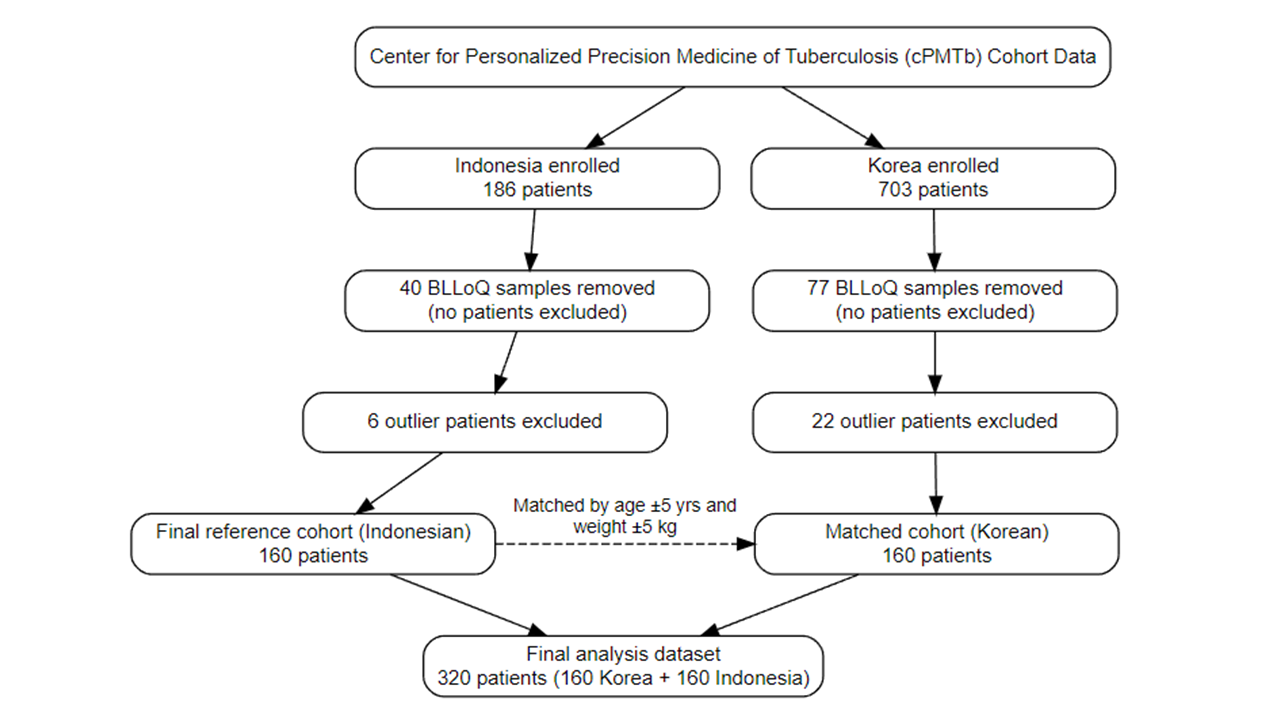

Supplement: S2 Fig — This diagram illustrates the selection process from the Center for Personalized Precision Medicine of Tuberculosis (cPMTb) cohort. (TIF) [file pone.0340133.s002.tif]

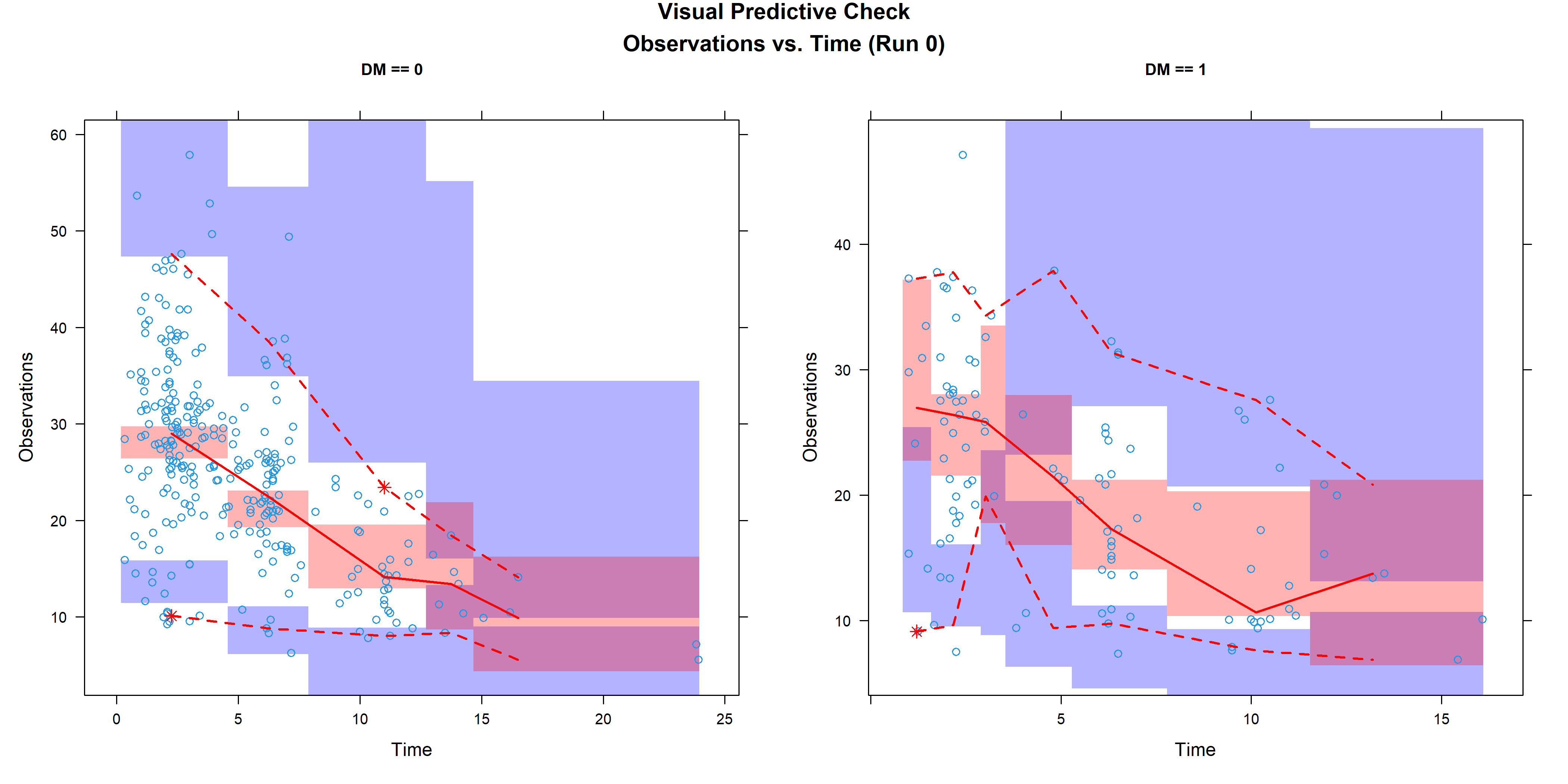

Supplement: S3 Fig — The left panel shows individuals without DM (DM == 0), and the right panel shows individuals with DM (DM == 1). Open blue circles represent observed plasma concentrations. The solid red line indicates the observed median, and the dashed red lines represent the 5th and 95th percentiles of the observed data. Shaded areas represent the 90% prediction intervals (red for median, blue for outer percentiles) from 1,000 simulations using the final model. (TIFF) [file pone.0340133.s003.tiff]

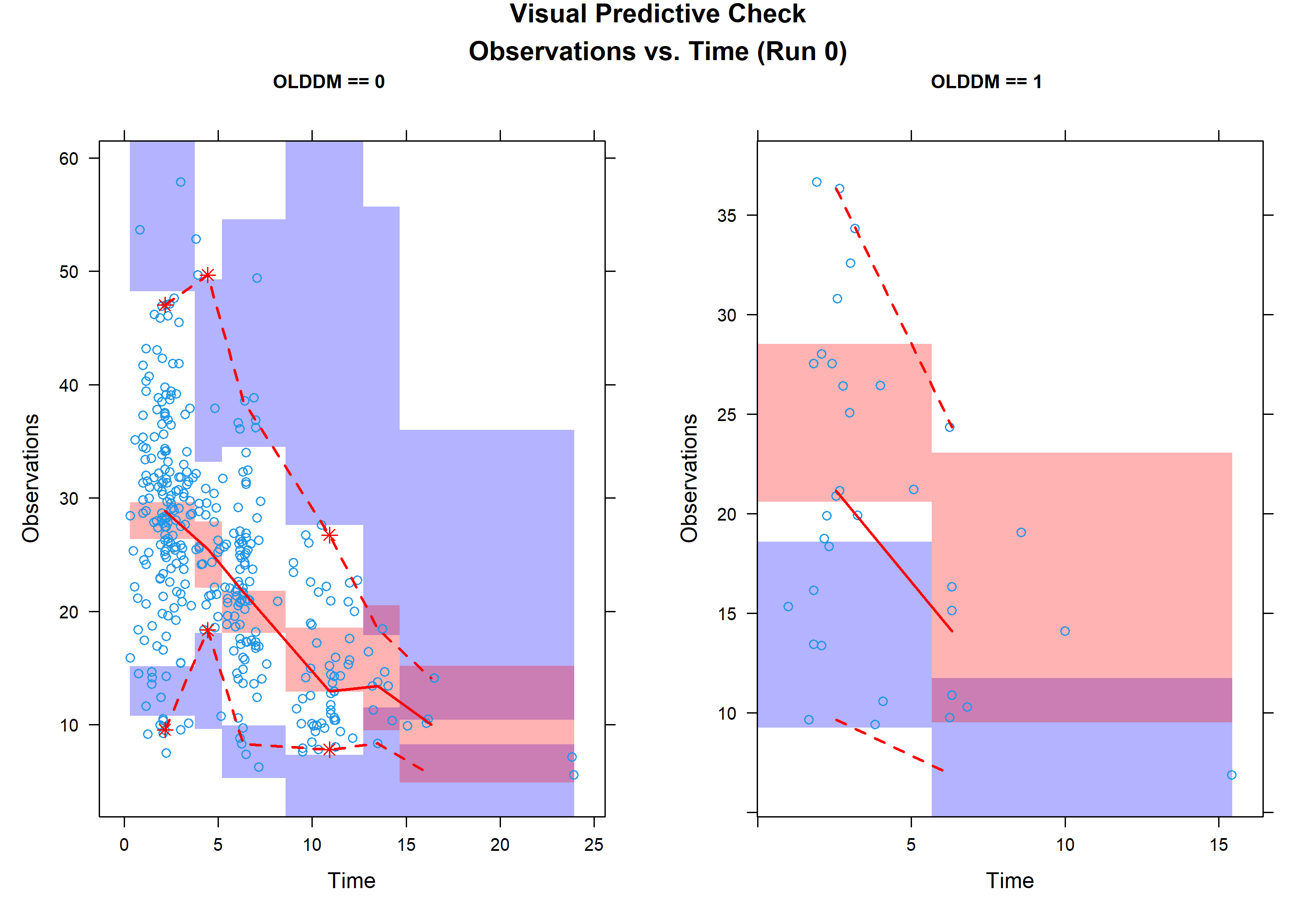

Supplement: S4 Fig — The left panel (DM == 0) includes all patients who are not both aged > 60 years and diagnosed with DM, while the right panel (DM == 1) includes elderly patients with DM (defined as age > 60 years with a DM diagnosis). Open blue circles represent observed plasma concentrations. The solid red line indicates the observed median, and the dashed red lines represent the 5th and 95th percentiles of the observed data. Shaded areas represent the 90% prediction intervals (red for median, blue for outer percentiles) from 1,000 simulations using the final model. (TIFF) [file pone.0340133.s004.tiff]

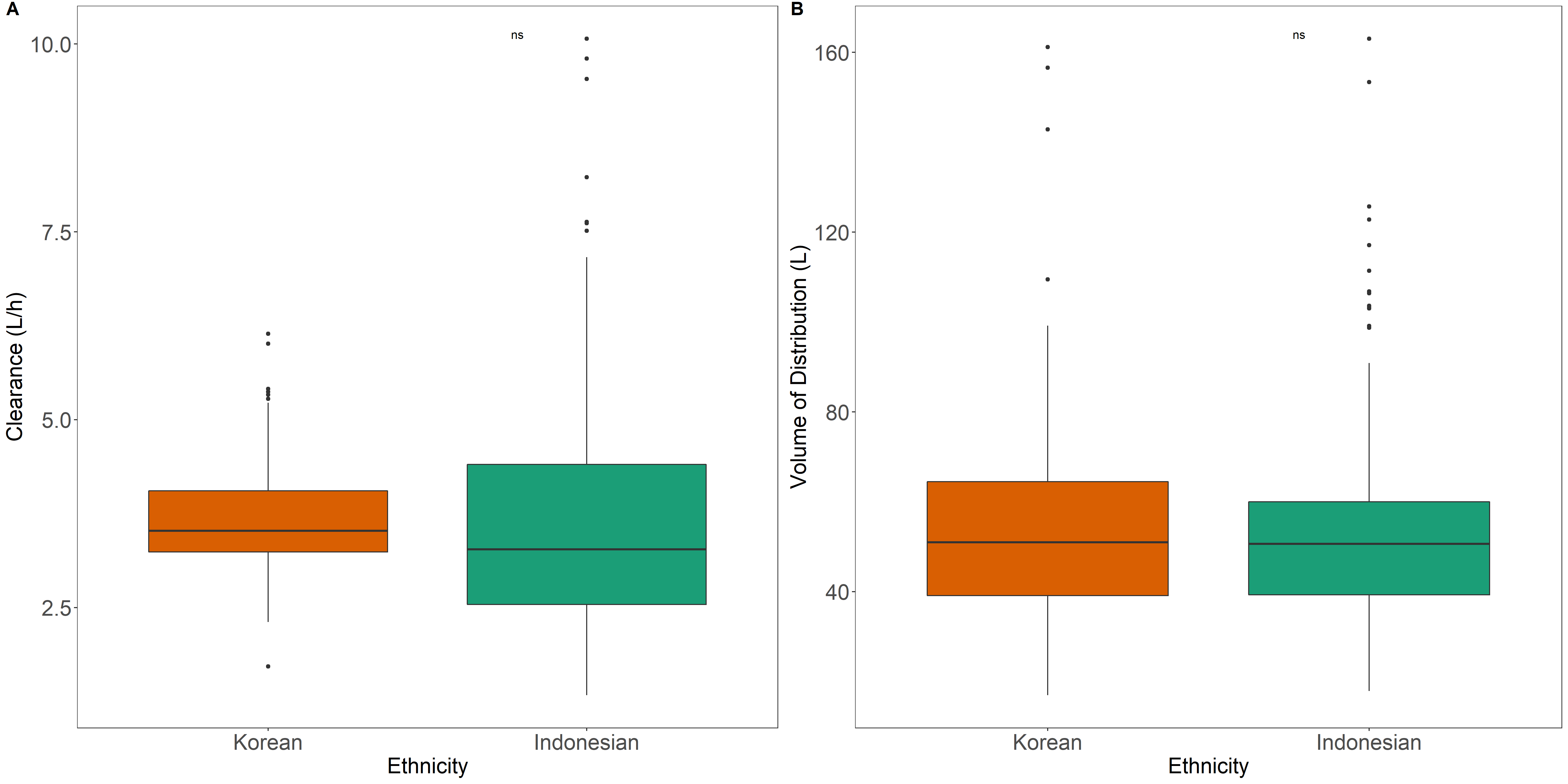

Supplement: S5 Fig — (A) Apparent clearance among Korean-Indonesian ethnicities. (B) Apparent volume of distribution among Korean-Indonesian ethnicities Box plot showing the interquartile range of each PK parameter. The groups are represented as follows: orange, Korean; green, Indonesian. The straight line in the upper part of the box plot represents the t-test results. **** P < 0.001, ns: non-significant). (TIFF) [file pone.0340133.s005.tiff]
