# Supplementary material for: Population pharmacokinetics model of pyrazinamide to optimize tuberculosis treatment: An interethnic cohort study of diabetes mellitus effect on drug exposure
Source: PLoS One. 2026 Jan 29;21(1):e0340133. doi: 10.1371/journal.pone.0340133 (PMC12854426; doi:10.1371/journal.pone.0340133)
Supplement: S1 Table — (DOCX) [file pone.0340133.s006.docx]

**S1 Table. Covariate selection process of other potential models.**

| **Forward Inclusion** | **Characteristics** | **Minimization** | **△OFV** | **Degree of Freedom** | ***p* value** | **Issue** | **Wald test** |  |
| --- | --- | --- | --- | --- | --- | --- | --- | --- |
|  |  |  |  |  |  |  |  |  |
|  | LBW and DM on CL/F_Indonesia_  LBW and OLDDM on CL/F_Korean_  LBW on Vd/F | successful | 25.66 | 4 | 4E-05 |  | Pass |  |
|  | LBW and OLDDM on CL/F_Indonesia_  LBW and OLDDM on CL/F_Korean_  LBW on Vd/F | successful | 25.26 | 4 | 4E-05 | High SE | Fail |  |
|  | LBW and DM on CL/F_Indonesia_  LBW and DM on CL/F_Korean_  LBW on Vd/F | successful | 28.45 | 4 | 1E-05 |  | Pass |  |
|  | LBW and OLDDM on CL/F_Indonesia_  LBW and DM on CL/F_Korean_  LBW on Vd/F | successful | 28.04 | 4 | 1E-05 | High SE | Fail |  |
|  |  |  |  |  |  |  |  |  |
| OFV, objective function value; ${CL}/F,$ apparent clearance; ${V_{d}}/F,$ apparent volume of distribution; $K_{a},$ absorption rate constant; LBW, lean body weight; DM, diabetes mellitus; SE, standard error. | | | | | | | | |
