# Supplementary material for: Population pharmacokinetics model of pyrazinamide to optimize tuberculosis treatment: An interethnic cohort study of diabetes mellitus effect on drug exposure
Source: PLoS One. 2026 Jan 29;21(1):e0340133. doi: 10.1371/journal.pone.0340133 (PMC12854426; doi:10.1371/journal.pone.0340133)
Supplement: S2 Table — (DOCX) [file pone.0340133.s007.docx]

**S2 Table. Pharmacokinetics parameters of pyrazinamide in both ethnicities**

| Parameters | | **Korean** | | | **Indonesian** | | |
| --- | --- | --- | --- | --- | --- | --- | --- |
|  |  | Old DM | Other | Total | DM | Non-DM | Total |
| C_max_ (mg/L) | | 21.46  (19.76 - 24.55) | 25.83  (21.9 - 31.72) | 25.62  (21.66 - 31.22) | 25.27  (21.13 - 32.09) | 28.70  (25.34 - 36.28) | 27.60  (23.47 - 33.12) |
| AUC_0-24h_ (mg.h/L) | | 261.4  (255.2 - 287.8) | 345.7  (309.2 - 374) | 343.2  (299.6 - 372.5) | 322.2  (216.8 - 423.4) | 388.6  (318.1 - 515.4) | 371.1  (270.2 - 466.9) |
| CL/F (L/h) | 4.61  (4.21 – 4.7) | | 3.47  (3.22 – 3.91) | 3.52  (3.25 – 4.05) | 3.74  (2.84 – 5.47) | 3.12  (2.36 – 3.8) | 3.23  (2.61 – 4.44) |
| Vd/F (L) | 56.72  (49.78 – 67.93) | | 50.60  (38.18 – 64.02) | 50.83  (38.44 - 64.33) | 51.27  (42.20 - 61.52) | 48.29  (38.12 – 57.82) | 50.28  (39.11 - 59.69) |
| Data are given as median and numbers in parentheses are interquartile range for the value of parameters. C_max_ and AUC_0-24h_ data were normalized with 1200 mg. C_max_: maximum plasma concentration, AUC_0-24h_: area under the concentration-time curve from 0 to 24 h, CL/F: apparent clearance; Vd/F: apparent volume of distribution. | | | | | | | |
