# Supplementary material for: Population pharmacokinetics model of pyrazinamide to optimize tuberculosis treatment: An interethnic cohort study of diabetes mellitus effect on drug exposure
Source: PLoS One. 2026 Jan 29;21(1):e0340133. doi: 10.1371/journal.pone.0340133 (PMC12854426; doi:10.1371/journal.pone.0340133)
Supplement: S3 Table — (DOCX) [file pone.0340133.s008.docx]

**S3 Table. Probability target attainment of simulated TB patients using WHO dose recommendation achieving a target AUC_0–24_ of 363 mg·h/L for both ethnicities.**

| WHO Dosing Recommendation | Indonesian | | | Korean | | | Mean ± Standard Deviation of Virtual Population |
| --- | --- | --- | --- | --- | --- | --- | --- |
|  | All | DM | Non-DM | All | Old DM | Other Patients |  |
| 800 mg  (<40 kg) | 70.4% | 65.0% | 75% | 70.4% | 69.4% | 71.5% | 34.75 ± 1.1 kg |
| 1200 mg  (40-54 kg) | 95.6% | 93.6% | 97.8% | 94.5% | 92.2% | 96.5% | 48.02 ± 5.1 kg |
| 1600 mg  (55-70 kg) | 99.2% | 98.8% | 99.6% | 99.4% | 96.8% | 99.5% | 59.8 ± 4.3 kg |
| 2000 mg  (>70 kg) | 96.3% | 94.7% | 97.9% | 94.4% | 92.6% | 96.2% | 76.53 ± 5.63 kg |
| DM: Diabetes Mellitus, Old DM: patient who aged > 60 years old with diabetes mellitus, AUC_0-24_: area under concentration curve from 0 to 24 hours | | | | | | | |
