# Supplementary material for: Population pharmacokinetics model of pyrazinamide to optimize tuberculosis treatment: An interethnic cohort study of diabetes mellitus effect on drug exposure
Source: PLoS One. 2026 Jan 29;21(1):e0340133. doi: 10.1371/journal.pone.0340133 (PMC12854426; doi:10.1371/journal.pone.0340133)
Supplement: S4 Table — (DOCX) [file pone.0340133.s009.docx]

**S4 Table. Probability target attainment of TB patients using simulated optimal dose achieving a target AUC_0–24_ of 363 mg·h/L for both ethnicities.**

| Body Weight | Simulated Dose | Indonesian (%) | | Korean (%) | |
| --- | --- | --- | --- | --- | --- |
|  |  | DM | Non-DM | Old DM | Other Patients |
| <40 kg | 1000 mg | 92.1 | 95.4 | 89 | 94.2 |
|  | 1250 mg | 99.1 | 99.6 | 94 | 99.4 |
|  | 1500 mg | 99.8 | 99.9 | 99.9 | 99.9 |
|  | 2000 mg | 99.9 | 99.9 | 99.9 | 99.9 |
|  | 2500 mg | 100 | 100 | 100 | 100 |
|  | 3000 mg | 100 | 100 | 100 | 100 |
| 40-54 kg | 1000 mg | 81.2 | 87.8 | 76.2 | 85.6 |
|  | 1250 mg | 96.9 | 98.4 | 94.3 | 97.8 |
|  | 1500 mg | 99.5 | 99.8 | 99.4 | 99.7 |
|  | 2000 mg | 99.9 | 99.9 | 99.9 | 99.9 |
|  | 2500 mg | 99.9 | 100 | 99.9 | 99.9 |
|  | 3000 mg | 100 | 100 | 100 | 100 |
| 55-70 kg | 1000 mg | 63.7 | 72.5 | 57 | 62.3 |
|  | 1250 mg | 86.4 | 89.4 | 83 | 85 |
|  | 1500 mg | 95.8 | 98.8 | 94.1 | 97 |
|  | 2000 mg | 99.9 | 99.9 | 99.9 | 99.9 |
|  | 2500 mg | 99.9 | 99.9 | 99.9 | 99.9 |
|  | 3000 mg | 100 | 100 | 100 | 100 |
| >70 kg | 1000 mg | 42.2 | 52 | 38 | 48.4 |
|  | 1250 mg | 79 | 83 | 73 | 83 |
|  | 1500 mg | 85.5 | 88.5 | 84.5 | 89.5 |
|  | 2000 mg | 94.7 | 97.9 | 92.6 | 96.2 |
|  | 2500 mg | 99.7 | 99.9 | 99.6 | 99.9 |
|  | 3000 mg | 100 | 100 | 99.9 | 100 |
| DM: Diabetes Mellitus, Old DM: patient who aged > 60 years old with diabetes mellitus, AUC_0-24_: area under concentration curve from 0 to 24 hours | | | | | |
