# Supplementary material for: Population pharmacokinetics model of pyrazinamide to optimize tuberculosis treatment: An interethnic cohort study of diabetes mellitus effect on drug exposure
Source: PLoS One. 2026 Jan 29;21(1):e0340133. doi: 10.1371/journal.pone.0340133 (PMC12854426; doi:10.1371/journal.pone.0340133)
Supplement: S1 File — (PDF) [file pone.0340133.s010.pdf]

Human Participants Research Checklist

Complete the following if your study involved human participants or human participants' data. These questions should be addressed for prospective and retrospective studies.

1. Did you obtain ethics approval for this study?

   - If yes, please upload (file type "Other") the original approval document you received from your ethics committee. <u>If the original document is in another language, please also provide an English translation.</u>

     X Uploaded ___ N/A

   ⇒ Yes, the study was ethically approved by the IRB in Korea (Inje University Busan Paik Hospital) and Indonesia (Dr. Soetomo General Academic Hospital) (page 6 lines 113-116), and the approval letters are uploaded in the recommended file type (Other)

   - If you did not obtain ethical approval, please explain why this was not required below.

2. If you prospectively recruited human participants for the study – for example, you conducted a clinical trial, distributed questionnaires, or obtained tissues, data or samples for the purposes of this study, please report in the Methods:

   i. the day, month and year of the **start and end** of the recruitment period for this study.

   ii. whether participants provided informed consent, and if so, what type was obtained (for instance, written or verbal, and if verbal, how it was documented and witnessed). If your study included minors, state whether you obtained consent from parents or guardians. If the need for consent was waived by the ethics committee, please include this information.

   X Completed ___ N/A

   ⇒ The study period when the participants were recruited is reported in the study data and population section Page 6 lines 121 – 122, and the inclusion and exclusion criteria were also reported in Page 6 lines 127 – 130.

   ⇒ Written consent was obtained from each participant, and it was stated under ethical approval and patient consent statement section Page 6 lines 116 – 118.

   ⇒ The data were accessed for research purposes on November 17th, 2021, as stated on page 6, lines 122–123.

   ⇒ The authors had access to information that could identify individual participants during data collection to ensure accurate data recording and verification. The data were stored in a centralized and secure database (https://smart.cpmtb.kr/#/cohort/status) that provides real-

time data summaries and interactive features. Access to this database is restricted to authorized users only, ensuring confidentiality and compliance with ethical guidelines. We have clarified these details in the revised manuscript (page 6, lines 124-126).

3. If you are reporting a retrospective study of medical records or archived samples, please report in the Methods section:
    i. the day, month and year when the data were accessed for research purposes
    ii. whether authors had access to information that could identify individual participants during or after data collection

___ Completed X N/A
